# Circulating cell‐free DNA methylation‐based multi‐omics analysis allows early diagnosis of pancreatic ductal adenocarcinoma

**DOI:** 10.1002/1878-0261.13643

**Published:** 2024-04-01

**Authors:** Guochao Zhao, Ruijingfang Jiang, Ying Shi, Suizhi Gao, Dansong Wang, Zhilong Li, Yuhong Zhou, Jianlong Sun, Wenchuan Wu, Jiaxi Peng, Tiantao Kuang, Yefei Rong, Jie Yuan, Shida Zhu, Gang Jin, Yuying Wang, Wenhui Lou

**Affiliations:** ^1^ Department of Pancreatic Surgery, Cancer Center, Zhongshan Hospital Fudan University Shanghai China; ^2^ Envelope Health Biotechnology Co. Ltd., BGI‐Shenzhen China; ^3^ Department of Hepatobiliary Pancreatic Surgery Changhai Hospital Affiliated to Navy Medical University Shanghai China; ^4^ Department of Medical Oncology, Cancer Center, Zhongshan Hospital Fudan University Shanghai China; ^5^ The Fifth Affiliated Hospital of Southern Medical University Guangzhou China; ^6^ BGI Genomics BGI‐Shenzhen China; ^7^ Shenzhen Engineering Laboratory for Innovative Molecular Diagnostics BGI‐Shenzhen China

**Keywords:** cfDNA, liquid biopsy, machine learning, methylation, mutation, pancreatic ductal adenocarcinoma

## Abstract

Pancreatic ductal adenocarcinoma (PDAC) is a highly aggressive cancer with a 5‐year survival rate of 7.2% in China. However, effective approaches for diagnosis of PDAC are limited. Tumor‐originating genomic and epigenomic aberration in circulating free DNA (cfDNA) have potential as liquid biopsy biomarkers for cancer diagnosis. Our study aims to assess the feasibility of cfDNA‐based liquid biopsy assay for PDAC diagnosis. In this study, we performed parallel genomic and epigenomic profiling of plasma cfDNA from Chinese PDAC patients and healthy individuals. Diagnostic models were built to distinguish PDAC patients from healthy individuals. Cancer‐specific changes in cfDNA methylation landscape were identified, and a diagnostic model based on six methylation markers achieved high sensitivity (88.7% for overall cases and 78.0% for stage I patients) and specificity (96.8%), outperforming the mutation‐based model significantly. Moreover, the combination of the methylation‐based model with carbohydrate antigen 19‐9 (CA19‐9) levels further improved the performance (sensitivity: 95.7% for overall cases and 95.5% for stage I patients; specificity: 93.3%). In conclusion, our findings suggest that both methylation‐based and integrated liquid biopsy assays hold promise as non‐invasive tools for detection of PDAC.

## Graphical abstract

Our study performed parallel genomic and epigenomic profiling of plasma cfDNA from pancreatic cancer patients and healthy individuals. A diagnostic model with six methylation markers achieved high sensitivity (88.7%) and specificity (96.8%); performance was further improved when combined with CA19‐9 (sensitivity: 95.7%; specificity: 93.3%). Our findings suggest that integrated liquid biopsy approaches show potential as non‐invasive methods for detecting pancreatic cancer.

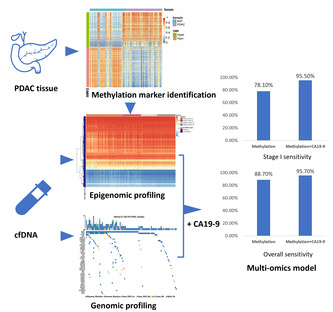

**Abbreviations**
AF: allele fractions
AUC: area under the curve
CA 19‐9: carbohydrate antigen 19‐9
cfDNA: circulating cell‐free DNA
CGI: CpG islands
CH: clonal hematopoiesis
ctDNA: circulating tumor DNA
CV: cross‐validation
DMR: differentially methylated regions
GO: Gene ontology
HMG: high mobility group
iAUC: integrated AUC
MeDEG: methylated‐differentially expressed genes
MPS: Methylation‐based prognostic scores
NAT: normal tissue adjacent to tumor
NGS: next‐generation sequencing
PDAC: pancreatic ductal adenocarcinoma
RFE: recursive feature elimination
ROC: Receiver operation characteristics
RRBS: reduced‐representation bisulfite sequencing
UMI: unique molecular identifier
UTRs: untranslated regions
WBC: white blood cells

## 1 Introduction

Pancreatic ductal adenocarcinoma (PDAC) is the major histological type of pancreatic cancer, characterized by its highly aggressive nature. It ranks as the sixth leading cause of cancer‐related death in China, with an overall 5‐year survival rate of 7.2% [1]. The poor prognosis was primarily due to diagnosis at an advanced stage and rapid progression. Since PDAC usually presents asymptomatic in its early stage, more than 85% of PDAC patients are diagnosed at advanced stages [2], precluding the possibility of curative surgical resection.

While serum carbohydrate antigen 19‐9 (CA19‐9) has been commonly used as a biomarker for PDAC diagnosis, its limited sensitivity in early‐stage patients and potential false positivity in non‐cancerous conditions limits its clinical utility for PDAC detection. Elevated CA19‐9 level was also observed in the patients with obstructive jaundice, other gastrointestinal tumors and even in healthy individuals [3]. A previous meta‐analysis reported a pooled sensitivity of 78.2% and a specificity of 82.8% for PDAC detection [4]. Therefore, there is a pressing need for novel detection approaches with improved performance.

Recent technological advances in detection of circulating tumor DNA (ctDNA), the tumor‐derived fraction of circulating cell‐free DNA (cfDNA), provided new opportunities for non‐invasive cancer diagnosis [5, 6]. As a biomarker, ctDNA harbors valuable genomic and epigenomic information of cancer, including sequence alteration, copy number variation, changes in methylation landscape, and cancer‐specific fragmentation patterns [6]. Recent evidence showed that simultaneous detection of multiple analytes in blood may enhance non‐invasive cancer detection [5, 6]. In this study, we aimed to identify novel markers in ctDNA and compared the performance of different analytes for PDAC detection.

## 2 Materials and methods

### 2.1 Study recruitment and sample collection

PDAC patients were recruited from the Zhongshan Hospital, Fudan University (cohort 1), and Changhai Hospital Affiliated to Navy Medical University (cohort 2). Healthy individuals were recruited from the Fifth Affiliated Hospital of Southern Medical University (cohort 3) and BGI (cohort 4) during March, 2019, to November, 2020. Written informed consent was obtained from all participants. The study design conformed to the standards set by the Declaration of Helsinki and was approved by the ethics committees of the Zhongshan Hospital (B2019‐297) and Shanghai Changhai Hospital ethics committees (CHEC2018‐039).

Blood was drawn before tumor resection or receiving anti‐tumor treatment for PDAC patients and at recruitment for healthy participants. PDAC tissue and normal tissue adjacent to tumor (NAT) for methylation sequencing were collected during surgery.

### 2.2 Library preparation and sequencing

Library preparation and sequencing approaches had been described previously [7]. Briefly, ultra‐deep targeted next‐generation sequencing (NGS) was conducted using a duplex unique molecular identifier strategy to suppress errors. A panel covering exons of 139 cancer driver genes (Table S1; Fig. S1), selected based on TCGA [8] and COSMIC [9] databases, was used. For targeted bisulfite sequencing, bisulfite‐treated single‐stranded DNA libraries were constructed and followed by enrichment using SeqCap Epi CpGiant Probes (Roche, Madison, WI, USA). The captured libraries were amplified and sequenced on MGISEQ‐2000 using 100 bp paired‐end sequencing.

### 2.3 Mutation‐based diagnostic models

Variants were called and filtered as described [7, 10]. Samples with targeted sequencing data were randomly divided into training set and testing set by a 7 : 3 split. PDAC diagnostic classifiers were built and validated using a vector machine algorithm by caret package in r [11].

### 2.4 Identification of differentially methylated regions (DMRs)

A Bayesian hierarchical model with smoothing was applied to 32 pairs of pancreatic cancer tissue and matched NAT to identify DMRs under the following criteria: the methylation difference between cancer and normal tissues > 0.2, region size ≥ 50 bp, containing ≥ 3 CpG sites, and ≥ 80% differentially methylated CpG sites [12]. DMRs were annotated and enriched using the r package of annotatr [13] and clusterprofiler [14], respectively.

### 2.5 Methylation‐based diagnostic models and DMR feature selection

A three‐step feature selection approach was applied to identify methylation‐related features. We first identified all relevant features by Boruta algorithm and then selected minimal‐optimal features using recursive feature elimination (RFE). DMR markers showing concordant changes between plasma samples and tissue samples were finally selected. Random forest models based on selected features were trained through 10‐fold cross‐validation (CV) in the training set and validated in the testing set.

To construct a multi‐omics model, random forest models combining mutational status and methylation were first trained and validated in cfDNA samples profiled with complete measurement. Secondly, to combine CA19‐9 levels with the cfDNA methylation‐based model, samples were predicted positive if either the methylation‐based model generated a positive prediction or the CA19‐9 levels was greater than 37.0 U·mL^−1^. The level of CA19‐9 was measured in each institute using COBAS e601 (Roche Diagnostic System, Basel, Switzerland) with Elecsys CA19‐9 reagents (Roche).

### 2.6 Statistical analyses

Wilcoxon‐Mann–Whitney test was used to compare differences between groups when the normality assumption was violated, and considered as statistically significant at a two‐sided *P*‐value < 0.05. Receiver operation characteristics (ROC) curves and corresponding area under the curve (AUC) were applied to assess the performance of diagnostic model. Statistical analyses and data visualization were performed using r statistical project (version 3.5.0) and python (version 3.7).

## 3 Results

### 3.1 Study design and participants

**Fig. 1:**
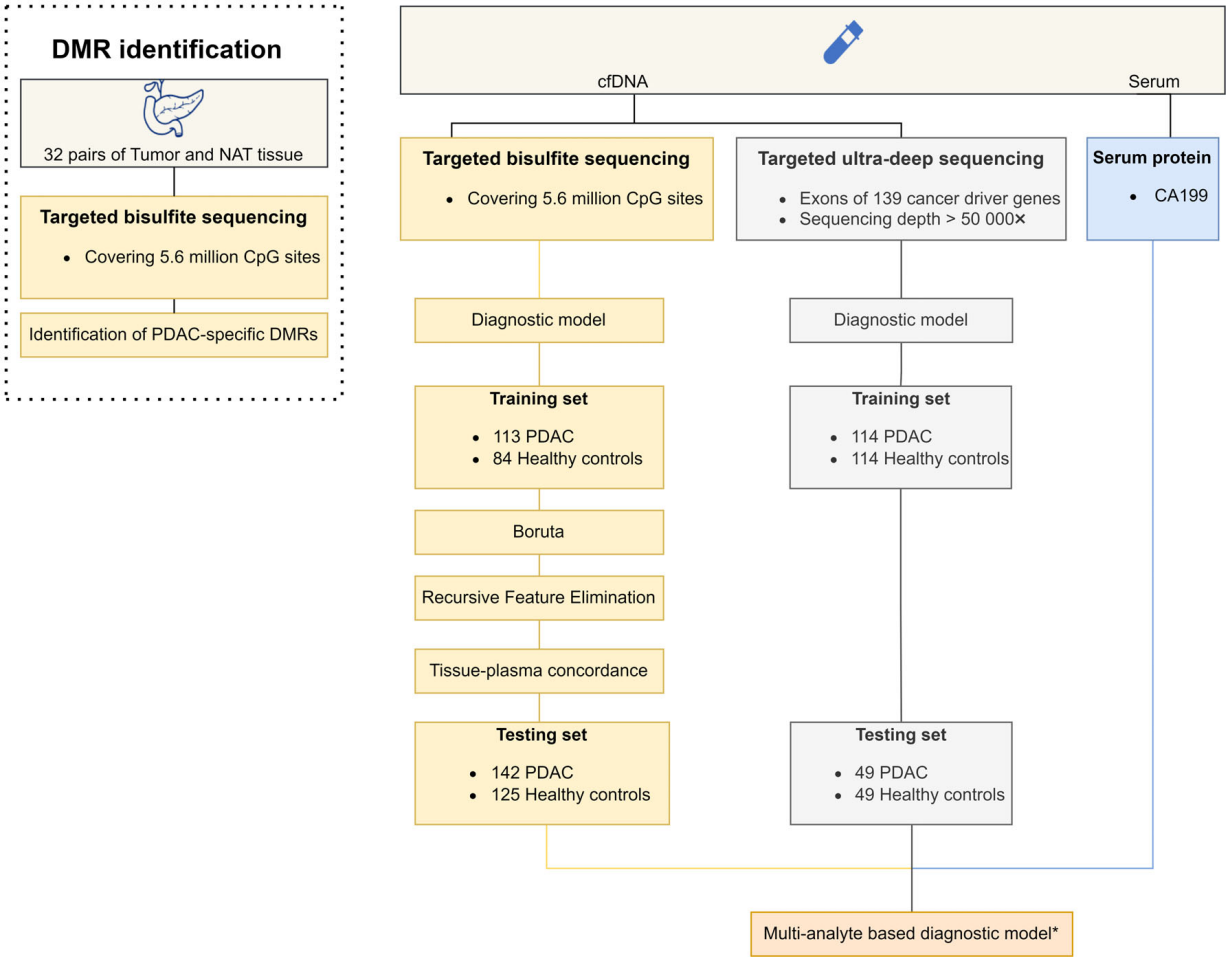
Flowchart of the study design. Analyses marked with asterisk were conducted in samples with complete measurements. CA19‐9, carbohydrate antigen 19‐9; cfDNA, circulating cell‐free DNA; DMR, differentially methylated regions; NAT, normal tissue adjacent to tumor; PDAC, pancreatic ductal adenocarcinoma.

In this study, we performed a comprehensive analysis of genomic and epigenomic alterations in plasma cfDNA of PDAC patients and healthy individuals (Fig. 1). Blood samples were collected from 262 PDAC patients (cohort 1: *n* = 169; cohort 2: *n* = 93) and 216 healthy controls (cohort 3: *n* = 84; cohort 2: *n* = 132). A slightly older median age was observed in PDAC patients (median = 64) than healthy controls (median = 58). Notably, 60% of the PDAC patients were diagnosed at early stages (AJCC stage 0‐II) (Table 1).

**Table 1:** Participants characteristics by assay type. Categorical variables were presented as number (percentage); Continuous variables were presented as median (interquartile range).

	Mutation		Methylation		Mutation + methylation		Total	
	PDAC	Healthy	PDAC	Healthy	PDAC	Healthy	PDAC	Healthy
Gender^a^								
Male	91 (55.83%)	69 (42.33%)	154 (60.39%)	86 (41.15%)	87 (55.77%)	67 (42.95%)	158 (60.31%)	88 (40.74%)
Female	72 (44.17%)	94 (57.67%)	101 (39.61%)	118 (56.46%)	69 (44.23%)	89 (57.05%)	104 (39.69%)	123 (56.94%)
Age	64 (56–69)	58 (53–64)	64 (57–69)	58 (53–64)	64 (56–69)	58 (53–64)	64 (57–69)	58 (53–64)
Stage								
0	2 (1.23%)		2 (0.84%)		2 (1.23%)		2 (0.75%)	
I	61 (37.42%)		76 (31.93%)		57 (35.19%)		80 (30.08%)	
II	35 (21.47%)		54 (22.69%)		35 (21.60%)		77 (28.95%)	
III	13 (7.98%)		33 (13.87%)		14 (8.64%)		34 (12.78%)	
IV	52 (31.90%)		73 (30.67%)		54 (33.33%)		73 (27.44%)	
CA19‐9	149.1 (39.75–598)	7.8 (5.08–11.39)	149.1 (39.75–598)	7.8 (5.08–11.39)	149.1 (39.75–598)	7.8 (5.08–11.39)	149.1 (39.75–598)	7.8 (5.08–11.39)

^a^ Gender information was missing for 5 healthy controls.

### 3.2 Mutation spectra of plasma cfDNA

To detect genomic sequence alterations, targeted ultra‐deep NGS was performed on plasma cfDNA extracted from 163 PDAC patients (all from cohort 1) and 163 healthy controls using a panel covering exons of 139 cancer driver genes [7]. A median de‐duplication depth of 4467X were achieved. In total, 242 mutations were detected in 113 (69.3%) PDAC patients and 56 mutations were identified in 48 (29.4%) healthy controls (Fig. S2).

**Fig. 2:**
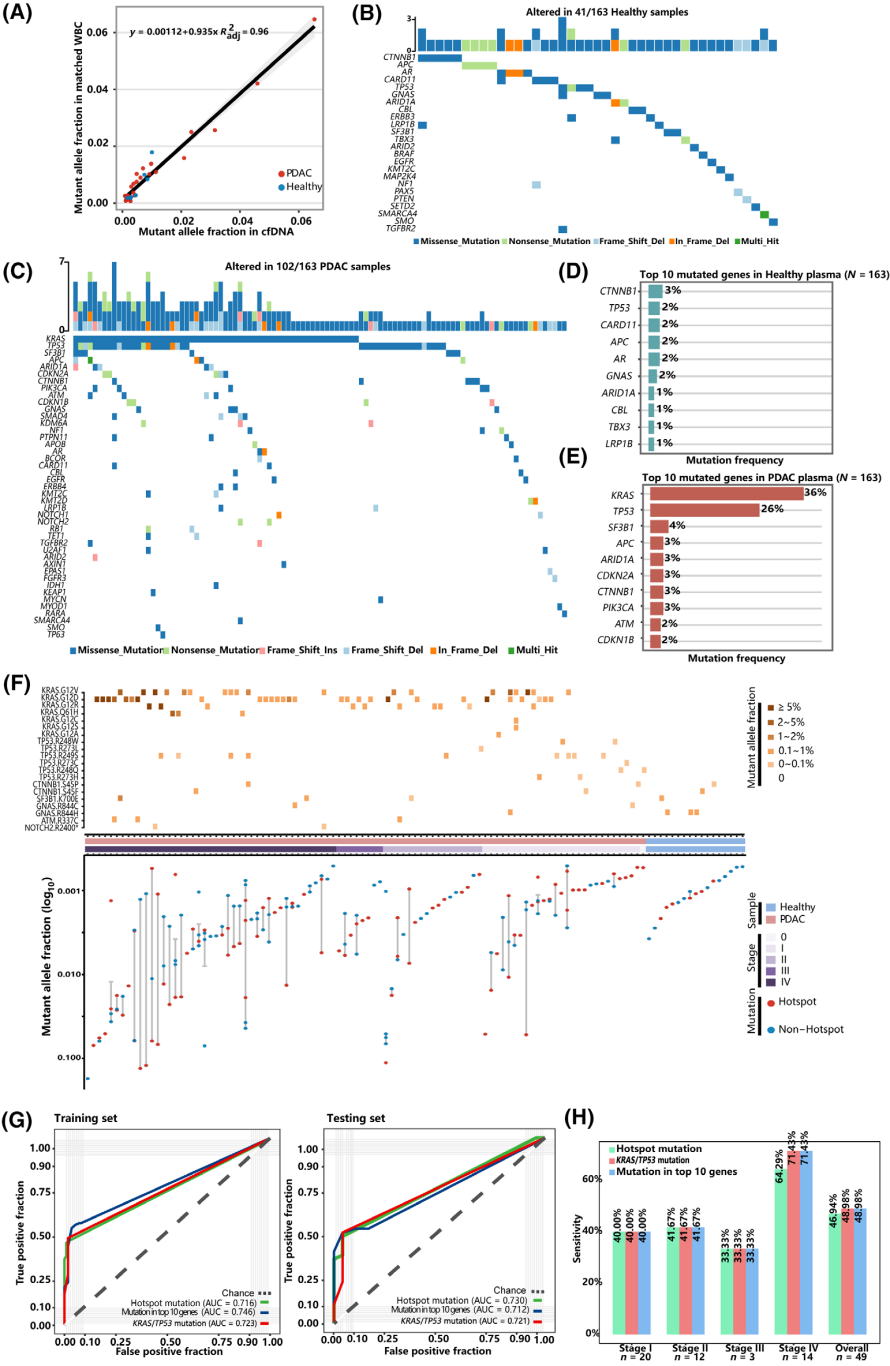
Mutation landscape of plasma cfDNA and mutation‐based diagnostic models for PDAC. (A) Correlation of AFs for shared mutations between cfDNA and paired WBC. Mutational landscape of plasma cfDNA in healthy controls (B) and (C) PDAC patients. Each column represents a PDAC or healthy plasma sample. Upper bar chart represents the number of mutations in each sample. Lower waterfall diagram depicts the mutated genes in each sample. Top 10 mutated genes in healthy (D) and PDAC plasma cfDNA (E). (F) The upper heatmap shows the mutant hotspots, and color depicts the level of mutant AFs. The Bottom plot demonstrates AFs of variants detected by hotspot status (G) Performance of the diagnostic models in the training (left) and testing (right) dataset using different indicators of mutational status. (H) PDAC sensitivity in the testing set by stage at the specificity of 95.9%. AF, allele fractions; cfDNA, circulating cell‐free DNA; PDAC, pancreatic ductal adenocarcinoma; WBC, white blood cells.

To control for the confounding effect of clonal hematopoiesis (CH) on cfDNA variant detection, we also sequenced gDNA of matched WBCs from cfDNA mutation‐positive participants. Shared non‐synonymous variants were found in 26 (23.0%) PDAC and 7 (14.6%) healthy participants with highly correlated allele fractions (AFs, Pearson *R* ^2^ = 0.96, Fig. 2A). The most frequently mutated genes were *TP53* (22.8%) and *GNAS* (11.4%). It highlighted the necessity of performing matched WBC sequencing when analyzing cfDNA variants in liquid biopsy assays.

After filtering for WBC‐shared variants, 212 variants remained in 102 (62.6%) PDAC cfDNA samples, with AFs ranging from 0.03% to 17.3% (median: 0.27%). *KRAS* (36%) and *TP53* (26%) were found to be the most frequently mutated genes in PDAC plasma cfDNA (Fig. 2C,E), which was consistent with the mutation spectrum in PDAC tissue from TCGA (Fig. S3). For healthy individuals, 49 mutations remained in 41 (25.1%) participants, with AFs ranging from 0.05% to 0.78% (median: 0.13%, Fig. 2B,D). Mutant AFs of cfDNA and tumor burden were significantly higher in PDAC than healthy controls. In PDAC patients, significantly higher AFs were observed in patient with greater tumor diameter, and in stage III‐IV patients (Fig. S4).

From the PDAC cfDNA variants, we identified 10 recurrent mutational hotspots [15] (Fig. 2F, Table S2), a pattern highly consistent with the PDAC mutational hotspots represented in the COSMIC database [9] (Fig. S5). In total, 75 PDAC patients (46.0%) harbored hotspot mutations, with the highest prevalence in *KRAS* p.G12 (*n* = 56) and *TP53* p.R249 (*n* = 9). A much lower fraction of healthy control plasma harbored these hotspot mutations (*n* = 7; 4.3%).

### 3.3 Diagnostic model based on mutation status

We next attempted to build a diagnostic classifier to distinguish PDAC from healthy plasma based on cfDNA mutational profile. In the training set (PDAC: *n* = 114, healthy control: *n* = 114), the model based on top 10 most frequently mutated genes generated an AUC of 0.746 (sensitivity: 53.3%, specificity: 96.5%; Fig. 2G). However, the top two mutated genes, *KRAS* and *TP53*, showed much higher feature importance (Fig. S6), leading to a comparable model based on these two genes with an AUC of 0.723 (sensitivity: 47.2%, specificity: 98.2%). Meanwhile, a model containing the top 10 recurrent hotspots achieved a similar performance, with an AUC of 0.716 (sensitivity: 43.9%, specificity: 99.1%). In the testing set (PDAC: *n* = 49, healthy control: *n* = 49), these three models achieved sensitivity of 48.9%, 49.0%, and 46.9%, respectively, at the same specificity of 95.9% (Fig. 2G). All models showed overall higher sensitivities in later stages than early stages (Fig. 2H). Overall, the classification models based solely on cfDNA mutation status had limited capability in differentiating PDAC and healthy plasma, especially for early‐stage PDAC.

### 3.4 Identification of PDAC‐associated epigenomic signatures

**Fig. 3:**
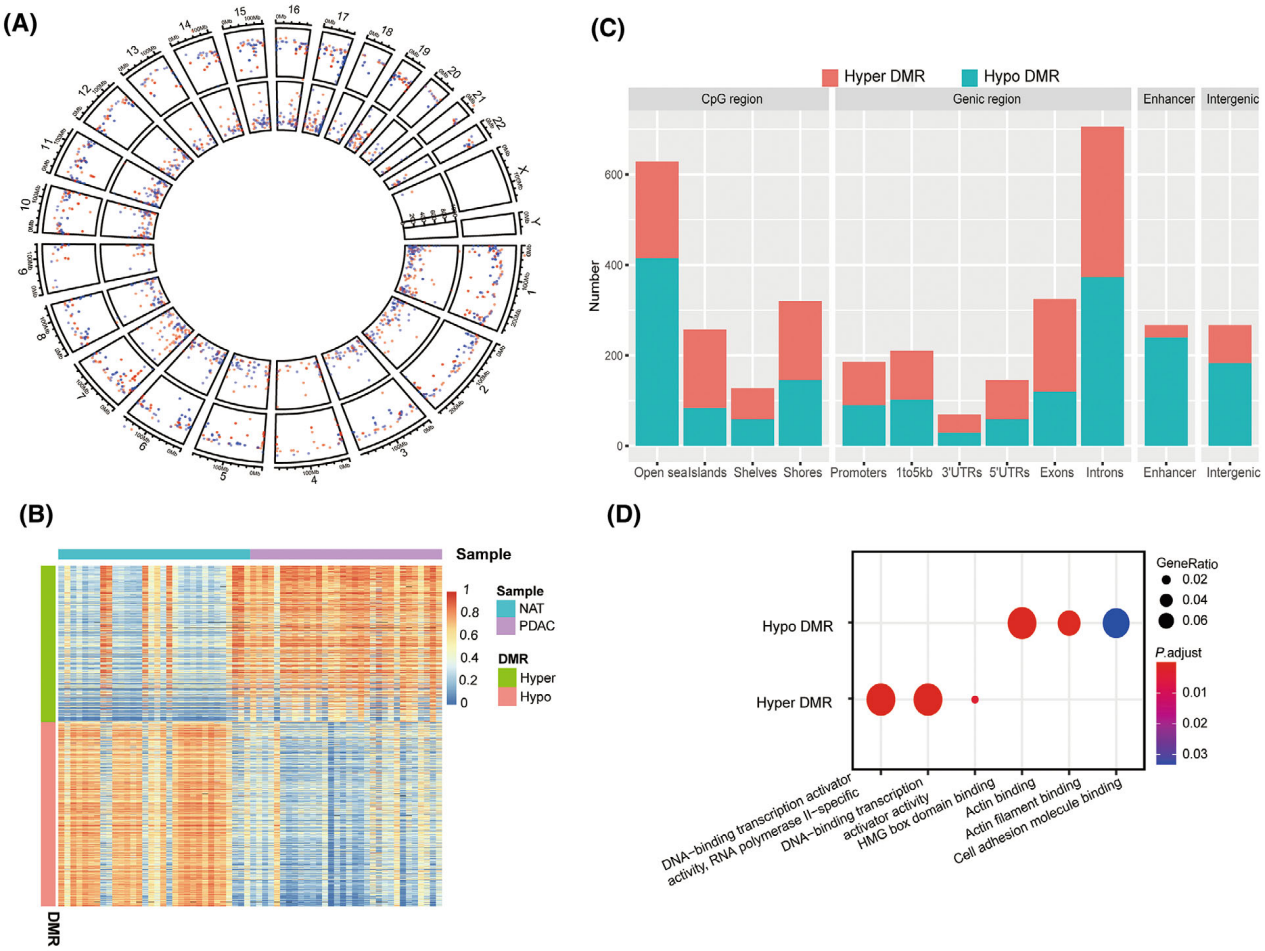
Differentially methylated regions (DMRs) discovered by targeted bisulfite sequencing of PDAC tumor and NAT tissues. (A) Circos plot showing the distribution of PDAC‐specific DMRs across the genome. Red points: hyper‐DMRs. Blue points: hypo‐DMRs. Circles from outer to inner circle were the overview of DMRs, the area statistics of hypermethylated regions, and hypomethylated regions, respectively. (B) Heatmaps showing DMR methylation levels in tissue data. (C) Locations of DMRs in genome. (D) GO term annotation of DMRs. DMR, differentially methylated regions; HMG, high Mobility Group; NAT, normal tissue adjacent to tumor; PDAC, pancreatic ductal adenocarcinoma; UTRs, untranslated regions.

To characterize epigenomic abnormalities associated with PDAC, we analyzed 5‐mC methylation profile of 32 pairs of PDAC and NAT using targeted bisulfite sequencing, covering 5.6 million of CpG sites genome‐wide. A total of 1173 DMRs were identified, with a median size of 208 bp (Fig. S7), of which 538 were hypermethylated DMRs (increased methylation in cancer tissue vs. normal; hyper‐DMRs) and 635 were hypomethylated DMRs (hypo‐DMRs) (Fig. 3A,B). These DMRs were annotated to various genic regions, with nearly 37% of the DMRs being annotated to introns, followed by exons (17.0%), intergenic (14.0%), the upstream of a transcriptional start site (11.0%), promoters (9.7%), 5′ untranslated regions (UTRs, 7.6%) and 3′ UTRs (3.7%). Annotation by CpG regions showed that hyper‐DMRs were more likely to be annotated to CpG islands (CGI), while hypo‐DMRs were more likely to be annotated to CpG open sea. Hypo‐DMRs were more likely to be enriched in enhancers than hyper‐DMRs (Fig. 3C). GO analysis of DMR‐associated genes revealed that hyper‐DMRs were significantly enriched for genes involved in transcription activation and high mobility group (HMG)‐box domain binding activity. Therefore, hypermethylation of these DMRs may initiate systematic transcriptional aberration in PDAC. On the other hand, hypo‐DMRs appeared to be significantly enriched for genes involved in actin binding and cell adhesion (Fig. 3D), possibly associated with the activation of fibroblasts/stromal cells in pancreatic tumors [16].

### 3.5 PDAC diagnostic models based on DMR markers

We next performed cfDNA methylation profiling for 255 PDAC cancer plasma and 209 healthy control plasma samples using targeted bisulfite sequencing. Notably different methylation patterns between PDAC and healthy plasma cfDNA were observed for 1173 DMRs identified from tissue analysis (Fig. S8). Interestingly, while the majority of hyper‐DMRs showed higher methylation levels in cancer plasma than in healthy plasma as expected, the majority of hypo‐DMRs surprisingly also showed increased methylation levels overall in cancer plasma. This observation was consistent with a previous report which showed that tissue‐derived methylation signature was abundant in plasma from cancer patients [17].

**Fig. 4:**
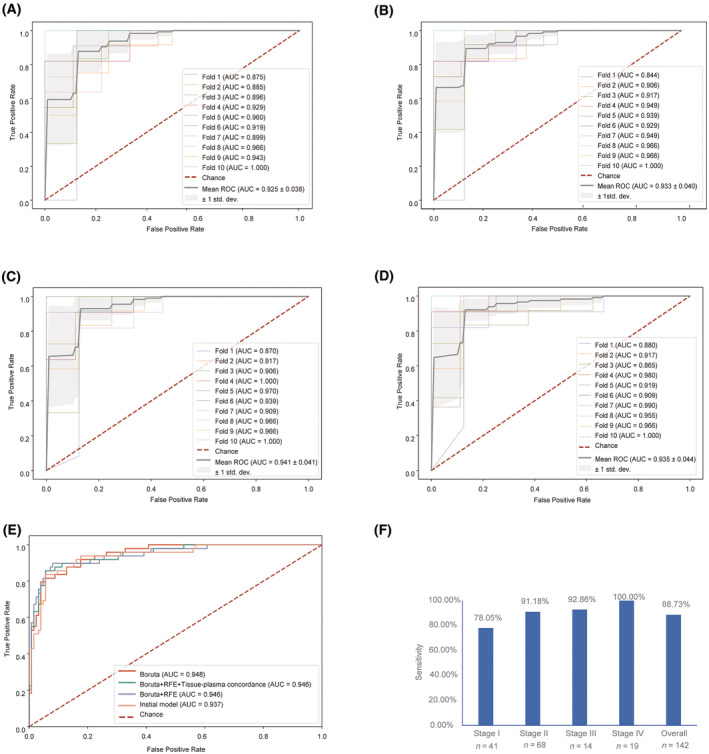
Methylation‐based PDAC diagnostic models. Performance of random forest model in the training set using all DMRs (A), and 200 DMRs selected by Boruta algorithm (B) as features. Performance in the training set using 13 DMRs selected by Boruta followed by RFE (C) and 6 DMRs in addition filtered by tissue‐plasma concordance (D). (E) Comparison of performance of the above 4 diagnostic models in the testing set. (F) PDAC sensitivity in the testing set by stage (specificity = 96.8%). AUC, area under the curve; RFE, recursive feature elimination; ROC, receiver operation characteristics.

Random forest models were then trained to classify PDAC plasma from healthy controls based on DMR methylation ratios. Among samples with targeted methylation sequencing data, 70% of randomly selected PDAC patients from cohort 1 (*n* = 113) and all healthy controls from cohort 3 (*n* = 84) were used as training set while the remaining samples were used as testing set. In the training set, 10‐fold CV achieved an AUC of 0.925 (Fig. 4A). A three‐step feature selection approach was then applied to select the optimal features. First, Boruta algorithm was used to prioritize all relevant features, and the resulting model based 200 selected features achieved a 10‐fold CV AUC of 0.933 (Table S3; Fig. 4B). To further select optimal features, the RFE process was utilized and 13 features were selected, resulting in a corresponding model with a 10‐fold CV AUC of 0.941 (Fig. 4C). Finally, as hypothesized, hypo‐DMR markers were more likely to be associated with stromal cells instead of cancerous cells and hence might be less specific for PDAC detection. Therefore, only hyper‐DMRs that showed concordant changes between plasma and tissue samples (Fig. S9) were considered. The final methylation‐based classification model was based on six tissue‐plasma concordant hyper‐DMRs (annotated to genes *KCNA3*, *PRRX*, *CCNA1*, *TRIM58*, and *NR2F1‐AS1*, Table S4) and achieved a 10‐fold CV AUC of 0.935 (Fig. 4D).

The above models were then separately validated using the testing set (remaining 49 PDAC patients from cohort 1, all 93 PDAC patients from cohort 2, and 125 healthy controls from cohort 4) and their AUCs were compared. The achieved AUCs were comparable in all three models, being 0.948 in the model after Boruta selection, 0.946 with additional RFE and 0.946 in the model utilizing tissue‐plasma concordant hyper‐DMRs only, while the number of features utilized in the last model was much fewer (Fig. 4E). Therefore, the last model was selected and generated a sensitivity of 88.7% for PDAC overall, at a specificity of 96.8%. Additionally, this model exhibited a sensitivity of 78.0% in stage I patients (Fig. 4F). The sensitivities increased along with the advancing of disease stages, and this was observed in both internal samples (PDACs from cohort 1) and external samples (PDACs from cohort 2) in the testing set (Fig. S10).

### 3.6 Multi‐omics model

**Fig. 5:**
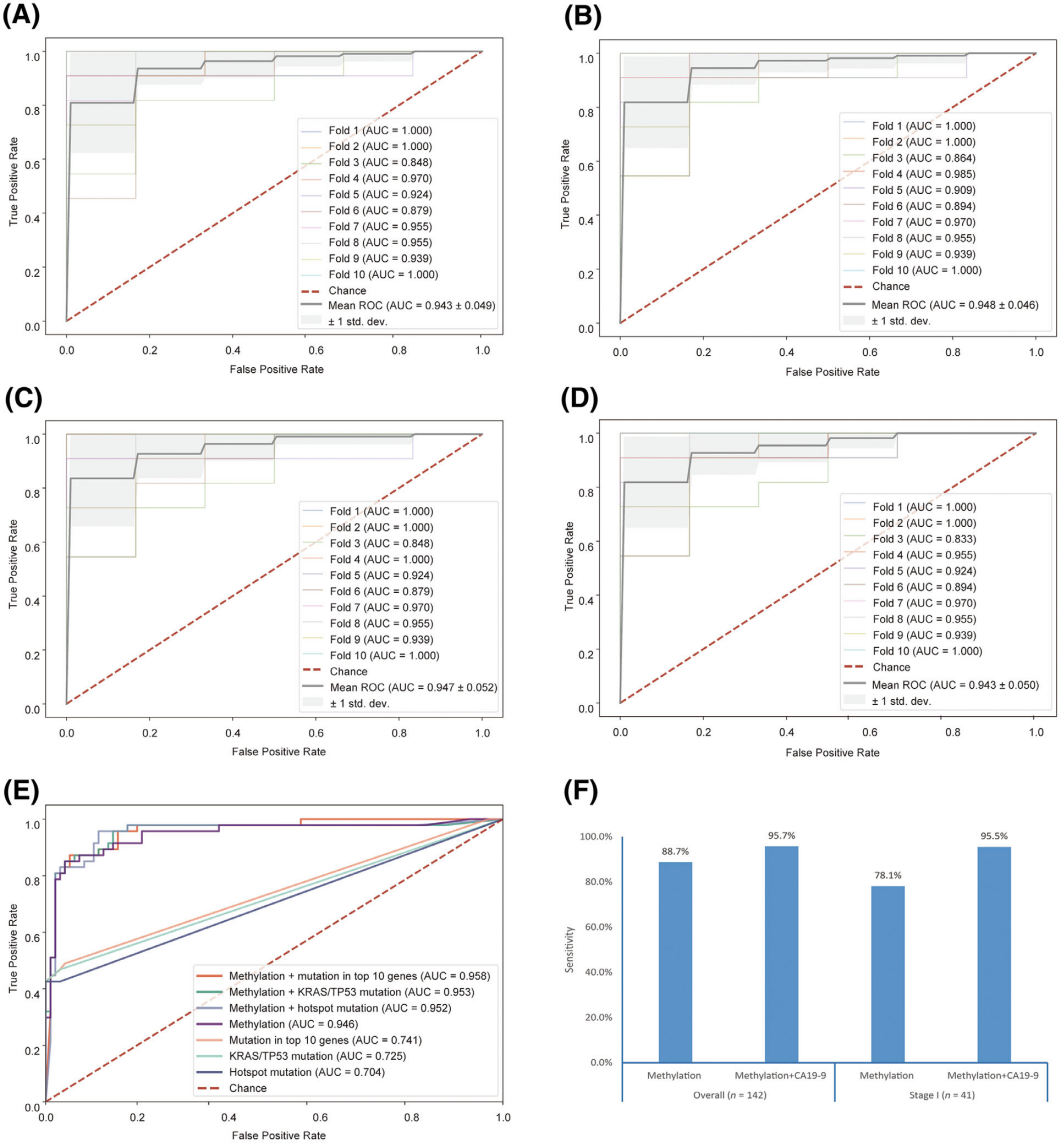
Multi‐omics based PDAC diagnostic models. (A) Performance of the diagnostic model in the training set based on 6 selected DMR markers. Performance of the diagnostic model in the training set based on methylation in combination with *KRAS*/*TP53* mutation (B) or mutation in top 10 genes (C) or hotspot mutation (D). (E) Performance in methylation‐based diagnostic model in the testing set with and without mutational status. (F) Sensitivities of diagnostic models in testing set based on different combination of analytes. AUC, area under the curve; CA19‐9, carbohydrate antigen 19‐9; RFE, recursive feature elimination; ROC, receiver operation characteristics.

Finally, we attempted to further improve the diagnostic performance by integrating the methylation data with the mutational status. We obtained both targeted sequencing and targeted bisulfite sequencing data for a total of 156 PDAC patients (training set: *n* = 109; testing set: *n* = 47) and 156 healthy controls (training set: *n* = 61; testing set: *n* = 95). The 10‐fold CV AUC slightly increased from 0.943 in the model based on methylation markers (Fig. 5A) to 0.948 by adding mutational status of the top two mutated genes (i.e., *KRAS* and *TP53*) (bi‐omics model I, Fig. 5B), and to 0.947 by adding mutational status of the top 10 mutated genes (bi‐omics model II) to the model (Fig. 5C). Adding mutational status of the top 10 hotspots to the methylation‐only model (bi‐omics model III) did not improve the performance (Fig. 5D). In the testing set, the methylation‐only model achieved an AUC of 0.946 while the bi‐omics model I achieved an AUC of 0.953 and bi‐omics model II achieved an AUC of 0.958 (Fig. 5E). At the specificity of 97.9% in the training set, both bi‐omics models I and II achieved a sensitivity of 80.9% and a specificity of 97.9%, compared to a sensitivity of 76.6% achieved by the methylation‐only model (Fig. S11). These results suggested that the bi‐omics models showed marginal performance enhancement over the methylation‐only model.

CA19‐9 is currently the most widely used biomarker for pancreatic cancer. We measured CA19‐9 for all PDAC patients as well as healthy controls from cohort 4 (*n* = 89). CA19‐9 alone, by applying the conventional diagnostic cutoff value of 37.0 U·mL^−1^ [18], achieved an overall sensitivity of 76.6% for PDAC patients (*n* = 142), a sensitivity of 77.3% in stage I patients (*n* = 41) and a specificity of 95.5% for healthy controls in the testing set. To further improve the performance, we integrated the methylation levels with CA19‐9 levels for prediction. Due to unavailability of CA19‐9 measurement in heathy controls in the training set, samples were predicted as positive if either methylation‐based model or CA19‐9 generated a positive result, achieving a sensitivity of 95.7% for PDAC at a specificity of 93.3% in the testing set. Notably, for stage I PDAC patients, sensitivity increased to 95.5%, a remarkable improvement compared to a sensitivity of 78.0% obtained by the methylation‐based model or 77.3% by CA19‐9 alone (Fig. 5F). Among PDAC patients with a negative CA19‐9 result (*n* = 33), 27 patients (81.8%) were predicted as positive by the methylation‐based model. These data suggest that combined measurements of top methylation markers and CA19‐9 levels may have the potential to provide superior diagnostic performance for PDAC detection.

## 4 Discussion

In this study, we conducted comprehensive genomic and epigenomic profiling using targeted sequencing to cfDNA of PDAC plasma and healthy individuals. The combination of duplex UMIs and ultra‐deep depth of over 80 000×, along with matched WBC sequencing, ensured high specificity of variant identification. The detection rate of *KRAS* gene (~ 36%) in cfDNA of PDAC patients was comparable to recently published studies using NGS [5, 6, 19], but lower than *KRAS* prevalence in TCGA tissue data (~ 90%), with half of the variants detected having an AF below 0.5%. These results suggested that a large fraction of plasma samples may harbor *KRAS* mutations with AF < 0.2%, below the limit of detection of our NGS assay. Notably, we also identified potentially oncogenic variants in cancer driver genes in healthy plasma cfDNA, and some were hotspot mutations as reported in a previous study [20]. The presence of oncogenic variants in cfDNA of asymptomatic individuals might be related to CH and somatic clonal expansion in normal tissues, which poses additional challenge to implementation of liquid biopsy. Further studies are needed to fully evaluate the background mutation burden in average‐risk individuals. Our results collectively indicated that PDAC detection based on mutation alone is likely to have limited sensitivity using the current state‐of‐art sequencing technology.

In the methylation‐based diagnostic model, we achieved better performance compared to the mutation‐only model, and stepwise feature selections allowed us to reduce the number of features utilized in the final model to only six DMR markers, while maintaining the performance in the testing test. This provided a minimal set of DMR markers for further validation and potential application in the clinical setting using more cost‐effective assay forms for detection, such as multiplexed quantitative PCR.

We also found that previously reported diagnostic markers varied across studies (Table S5). Two major reasons may underlie this discrepancy. First, various methods were used for detection of the methylation signal, including MeDIP‐seq [21], Illumina Infinium 450 k Array [22], and reduced‐representation bisulfite sequencing (RRBS) [23, 24]. Of these, MeDIP‐seq cannot detect methylation alterations at single base‐pair resolution. Infinium 450 k Array and RRBS suffered from lower genome coverage due to either a limited number of probes or bias towards CpG‐rich regions. In our study, a targeted bisulfite sequencing panel covering 5.6 m CpG sites was applied, allowing us to measure CpG methylation level at single base‐pair resolution with increased genomic coverage. Notably, of the 200 DMRs features selected by Boruta algorithm, 136 located outside CGIs, which would be missed in RRBS‐based methylation profiling. Secondly, difference in the study population may also contribute to the variation. Most previous studies were conducted in the Caucasian population, while the Asian population were underrepresented. Nevertheless, of the six markers selected in the final methylation‐based diagnostic model, two genes (*KCNA3* and *TRIM58*) were previously reported through TCGA data analysis [8], hence suggesting that different detection methods may robustly re‐discover DMR markers with high performance. Overall, the novel biomarkers identified through our study add a rich resource for future investigation of epigenomic abnormalities and regulation mechanisms for PDAC.

To further improve the diagnostic performance, we experimented with combining multiple analytes, including mutation, methylation and the widely used PDAC biomarker, CA19‐9. Compared with methylation‐only model, additionally incorporating mutational status only marginally increase the performance; on the other hand, combining methylation markers with CA19‐9 improved the overall PDAC sensitivity to 95.7% and stage I sensitivity to 95.5% in the testing set, while maintaining a specificity of 93.3% in healthy controls. Recently, several studies showed that simultaneous detection of multiple analytes in blood may potentially improve performance for PDAC detection. For example, one study showed that a combination of five methylation markers and *KRAS* mutation status generated a sensitivity of 68% at the specificity of 86% [22]; another study reported a sensitivity of 64% through simultaneous detection of *KRAS* mutation and CA19‐9 [25]; both were inferior to our results. Recently, two diagnostic models that combined methylation markers and CA19‐9 levels were reported, and both were close to observed performance of our assay. A diagnostic model based on 13 methylation markers and CA19‐9 level achieved a sensitivity of 82% for early‐stage PDAC at a specificity of 94% [24], yet the diagnostic performance of methylation markers alone was quite limited (sensitivity: 40%, specificity: 98%). Another model incorporating 185 methylation markers and CA19‐9 levels achieved a sensitivity of 92% for stage I PDAC at a specificity of 89%, while achieving a sensitivity of 75% in a CA19‐9 negative PDAC patients [26]; however, the number of methylation markers included were a lot more than those utilized in our model, which would most likely limit the clinical feasibility of such panel of markers. Nevertheless, these reports support the notion that combined detection of multiple analytes may complement each other and hence improve the overall performance.

Our study also had a couple of limitations. First, we did not evaluate benign lesion of pancreas in this study, and because such benign lesions have been reported to also show aberrant methylation and elevated CA19‐9 levels [27, 28], our models may need to be adjusted if used for differentiating PDAC from benign pancreatic lesions in clinical setting. In addition, measurement on CA19‐9 levels was unavailable for health controls in the training set and therefore participants were categorized as positive if either CA19‐9 levels or the methylation‐based model was tested positive. However, it provides the flexibility to integrate our diagnostic model with clinical standard of care, which might enhance the application in the clinical practice.

## 5 Conclusions

In conclusion, our results identified novel biomarkers for detection of PDAC by profiling genomic and epigenomic abnormalities of cfDNA through massive parallel sequencing. We also showed that performance of diagnostic models may be further improved by integrating methylation markers with the protein marker CA19‐9, resulting in remarkable detection sensitivity and specificity. Importantly, given only a handful of effective methylation markers and the conventional CA19‐9 test were utilized, such methodology may be potentially developed into a cost‐effective diagnostic assay. Our findings hold promise for the development of clinically valuable diagnostic tools for the improved management of PDAC.

## Conflict of interest

GZ, SG, WD, YZ, WW, TK, YR, JY, GJ and WL have declared no competing interest. RJ, YS, ZL, JS, JP, and YW are employees of Envelope Health Biotechnology Co. Ltd., BGI‐Shenzhen. SZ is an employee of BGI Genomics, BGI‐Shenzhen.

## Author contributions

WL and YW conceived and designed this study. GZ, SG, DW, YZ, WW, TK, YR, JY, GJ, and WL collected samples, clinical information and followed participants. ZL, JP and JS performed the experiments. RJ, and YS analyzed data. GZ, RJ, YS, SZ, YW, and WL wrote the manuscript. WL, YW and SZ provided intellectual discussions and ideas regarding the content of manuscript.

### Peer review

The peer review history for this article is available at https://www.webofscience.com/api/gateway/wos/peer‐review/10.1002/1878‐0261.13643.

## Supporting information


**Fig. S1.** Percent of TCGA samples predicted to be covered by the targeted sequencing panel utilized in the present study by different cancer types.
**Fig. S2.** cfDNA mutation landscape of PDAC and healthy plasma before filtering with WBCs‐shared variants.
**Fig. S3.** Comparison of mutation landscape of plasma cfDNA in present study with TCGA tissue data for PDAC patients.
**Fig. S4.** Genetic alterations by sample types and clinical characteristics.
**Fig. S5.** Frequencies of identified mutational hotspots in PDAC plasma samples.
**Fig. S6.** Feature importance of the top 10 most frequently mutated genes.
**Fig. S7.** Distribution of DMR lengths.
**Fig. S8.** Heatmaps showing methylation levels of 1173 DMRs in plasma cfDNA from PDAC and healthy controls, along with hierarchical clustering of DMRs.
**Fig. S9.** Boxplots showing distributions of cfDNA methylation levels for the 13 DMR markers identified by RFE in the feature selection process.
**Fig. S10.** Sensitivity of methylation‐based model in internal and external PDAC patients in testing set, respectively.
**Fig. S11.** Sensitivity for PDAC patients in testing set by different diagnostic models.

**Table S1.** The mutation panel covering 139 pan‐cancer driver genes.
**Table S2.** Hotspots detected in PDAC cfDNA dataset.
**Table S3.** DMR features selected by Boruta and in the following selection procedure.
**Table S4.** Gene annotation of selected DMRs in the final diagnostic model.
**Table S5.** Comparison among methylation‐based and integrated diagnostic models for PDAC.

## Data Availability

The datasets supporting the conclusions of this article are available in the CNGB Nucleotide Sequence Archive under accession number CNP0001894 (CNSA: https://db.cngb.org/cnsa).
