# Supplementary material for: Circulating cell‐free DNA methylation‐based multi‐omics analysis allows early diagnosis of pancreatic ductal adenocarcinoma
Source: Mol Oncol. 2024 Apr 1;18(11):2801–13. doi: 10.1002/1878-0261.13643 (PMC11547243; doi:10.1002/1878-0261.13643)
Supplement: Supplementary file 1 — Fig. S1. Percent of TCGA samples predicted to be covered by the targeted sequencing panel utilized in the present study by different cancer types. Fig. S2. cfDNA mutation landscape of PDAC and healthy plasma before filtering with WBCs‐shared variants. Fig. S3. Comparison of mutation landscape of plasma cfDNA in present study with TCGA tissue data for PDAC patients. Fig. S4. Genetic alterations by sample types and clinical characteristics. Fig. S5. Frequencies of identified mutational hotspots in PDAC plasma samples. Fig. S6. Feature importance of the top 10 most frequently mutated genes. Fig. S7. Distribution of DMR lengths. Fig. S8. Heatmaps showing methylation levels of 1173 DMRs in plasma cfDNA from PDAC and healthy controls, along with hierarchical clustering of DMRs. Fig. S9. Boxplots showing distributions of cfDNA methylation levels for the 13 DMR markers identified by RFE in the feature selection process. Fig. S10. Sensitivity of methylation‐based model in internal and external PDAC patients in testing set, respectively. Fig. S11. Sensitivity for PDAC patients in testing set by different diagnostic models. [file MOL2-18-2801-s002.pdf]

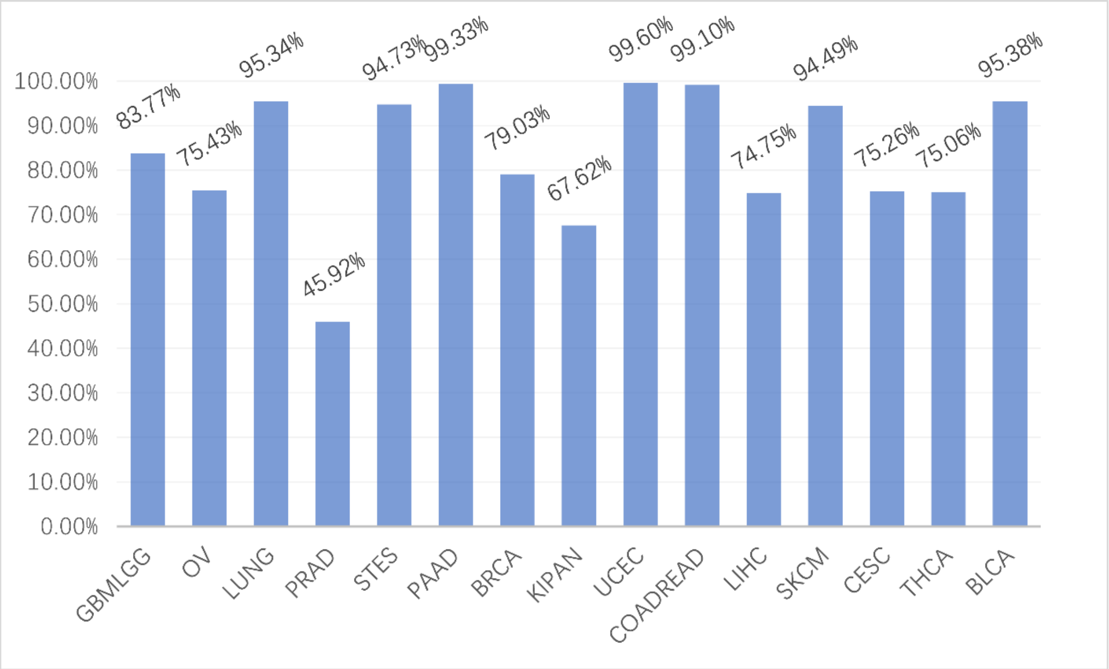

Figure S1. Percent of TCGA samples predicted to be covered by the targeted sequencing panel utilized in the present study by different cancer types. （GBMLGG: Glioblastoma multiforme and Brain Lower Grade Glioma; OV: Ovarian serous cystadenocarcinoma; LUNG: Lung adenocarcinoma; PRAD: Prostate adenocarcinoma; STADES: Stomach adenocarcinoma and Esophageal carcinoma; PAAD: Pancreatic adenocarcinoma; BRCA: Breast invasive carcinoma; KIPAN: Kidney pan- cancer; UCEC: Uterine Corpus Endometrial Carcinoma; COADREAD: Colon adenocarcinoma and Rectum adenocarcinoma; LIHC: Liver hepatocellular carcinoma; CESC: Cervical squamous cell carcinoma and endocervical adenocarcinoma; THCA: Thyroid carcinoma; BLCA: Bladder Urothelial Carcinoma）

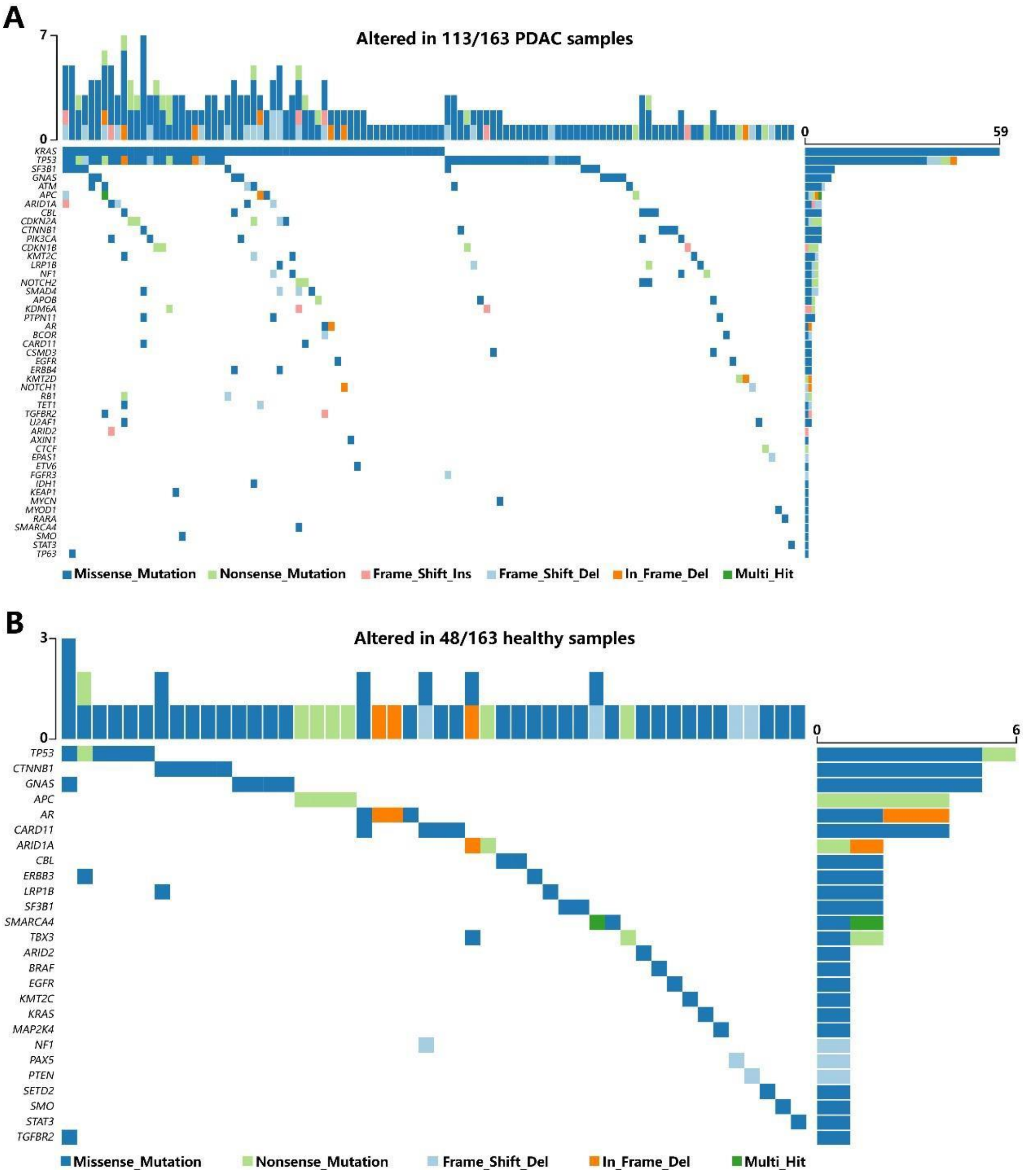

Figure S2. cfDNA mutation landscape of PDAC and healthy plasma before filtering with WBCs-shared variants. Each column represents a (*A*) PDAC or (*B*) healthy sample. Upper bar chart represents the number of mutations in each sample. Lower waterfall diagram depicts the mutated genes in each sample, and colors represent variant types.

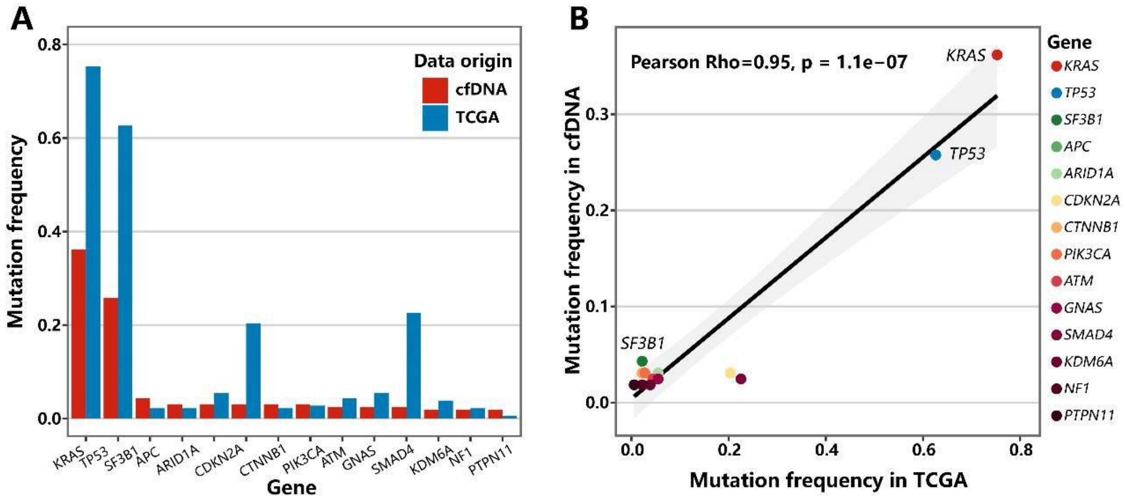

Figure S3. Comparison of mutation landscape of plasma cfDNA in present study with TCGA tissue data for PDAC patients. (*A*) Comparison of mutation frequency of top mutated genes identified in our study with TCGA data. (*B*) Correlation of mutation frequency of top mutated genes in our study with TCGA data.

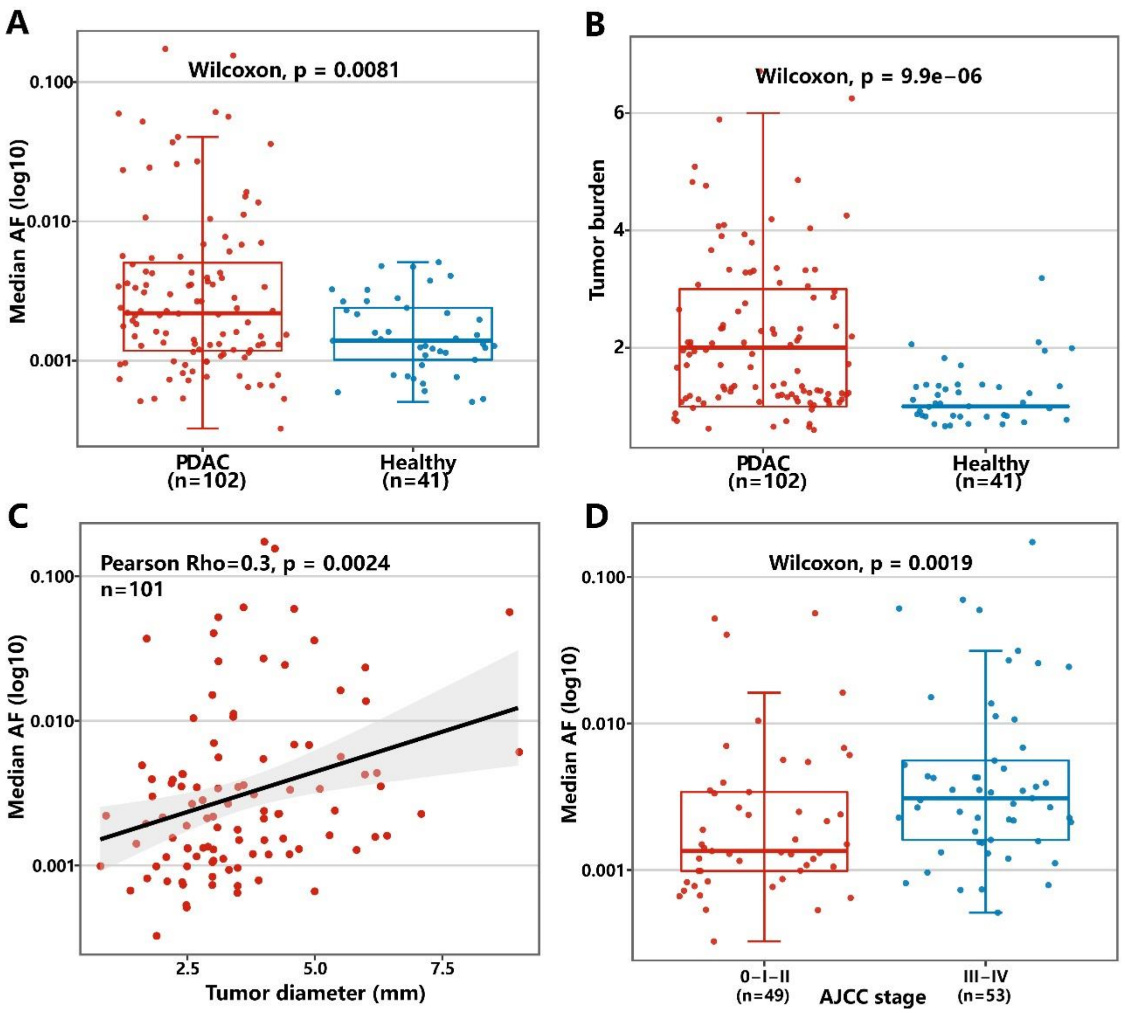

Figure S4. Genetic alterations by sample types and clinical characteristics. (*A*) Median mutant AFs and (*B*) tumor burden (defined as count of mutations identified) of PDAC and healthy plasma cfDNA. Only samples with detected variants were included in the analysis. (*C, D*) Correlation of median mutant AFs with tumor diameters (*C*), and tumor stages (*D*) in PDAC patients.

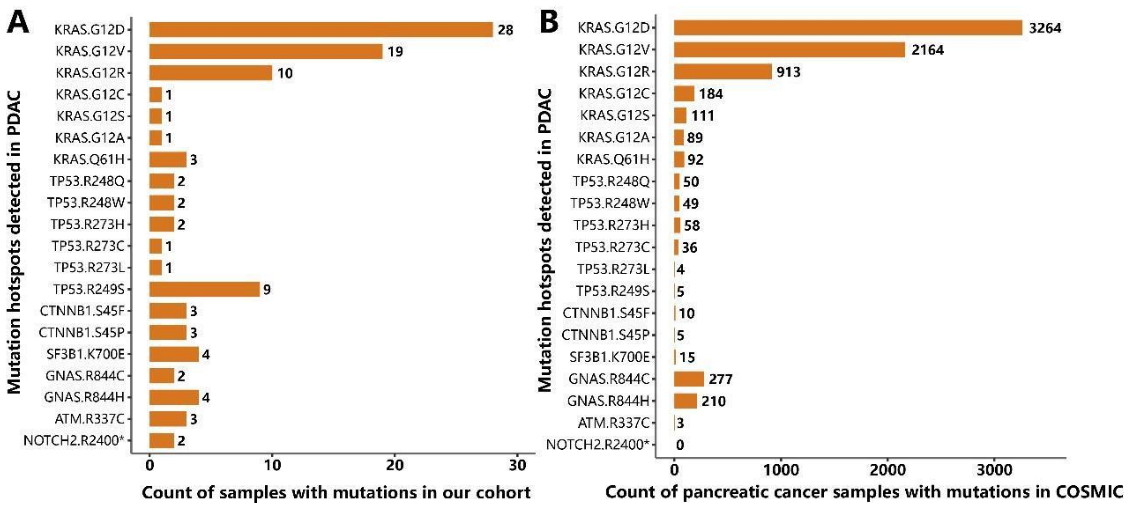

Figure S5. Frequencies of identified mutational hotspots in PDAC plasma samples. Count of (*A*) PDAC plasma samples and (*B*) pancreatic cancer samples from the COSMIC database carrying hotspot mutations.

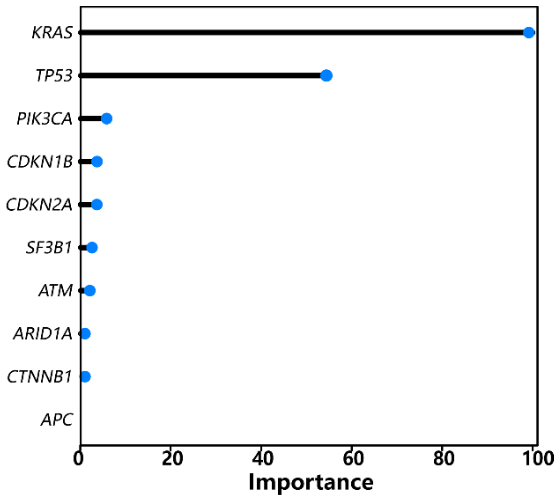

Figure S6. Feature importance of the top 10 most frequently mutated genes.

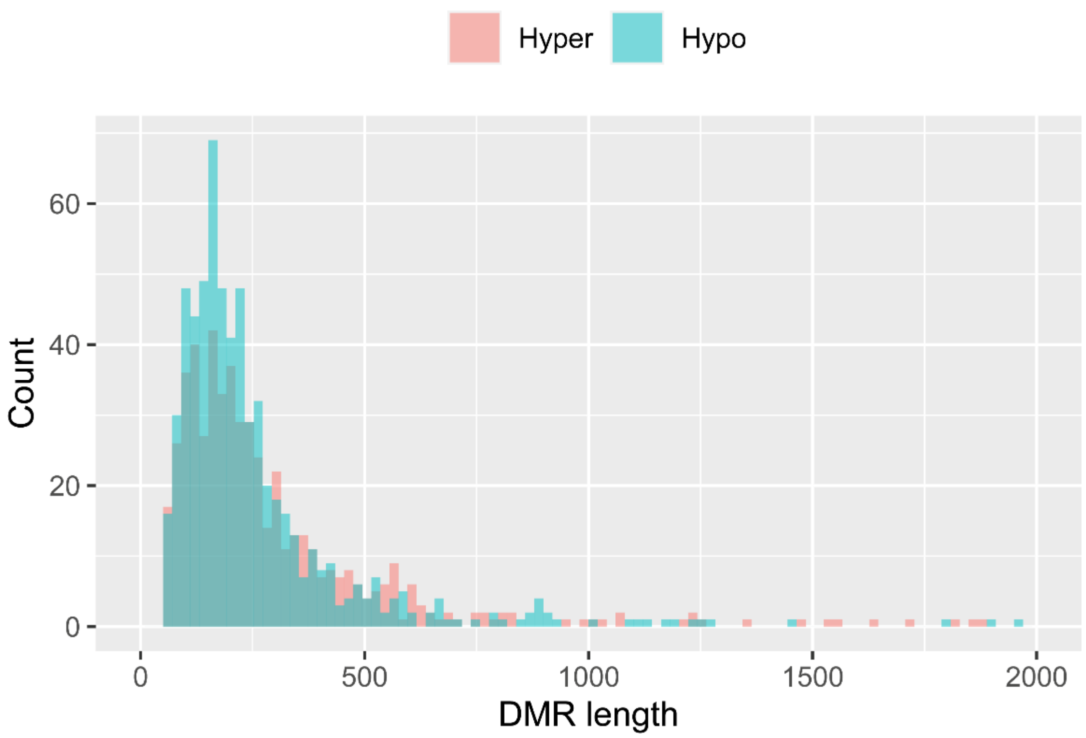

Figure S7. Distribution of DMR lengths. Pink and light turquoise denote hyper- and hypo-DMRs, respectively

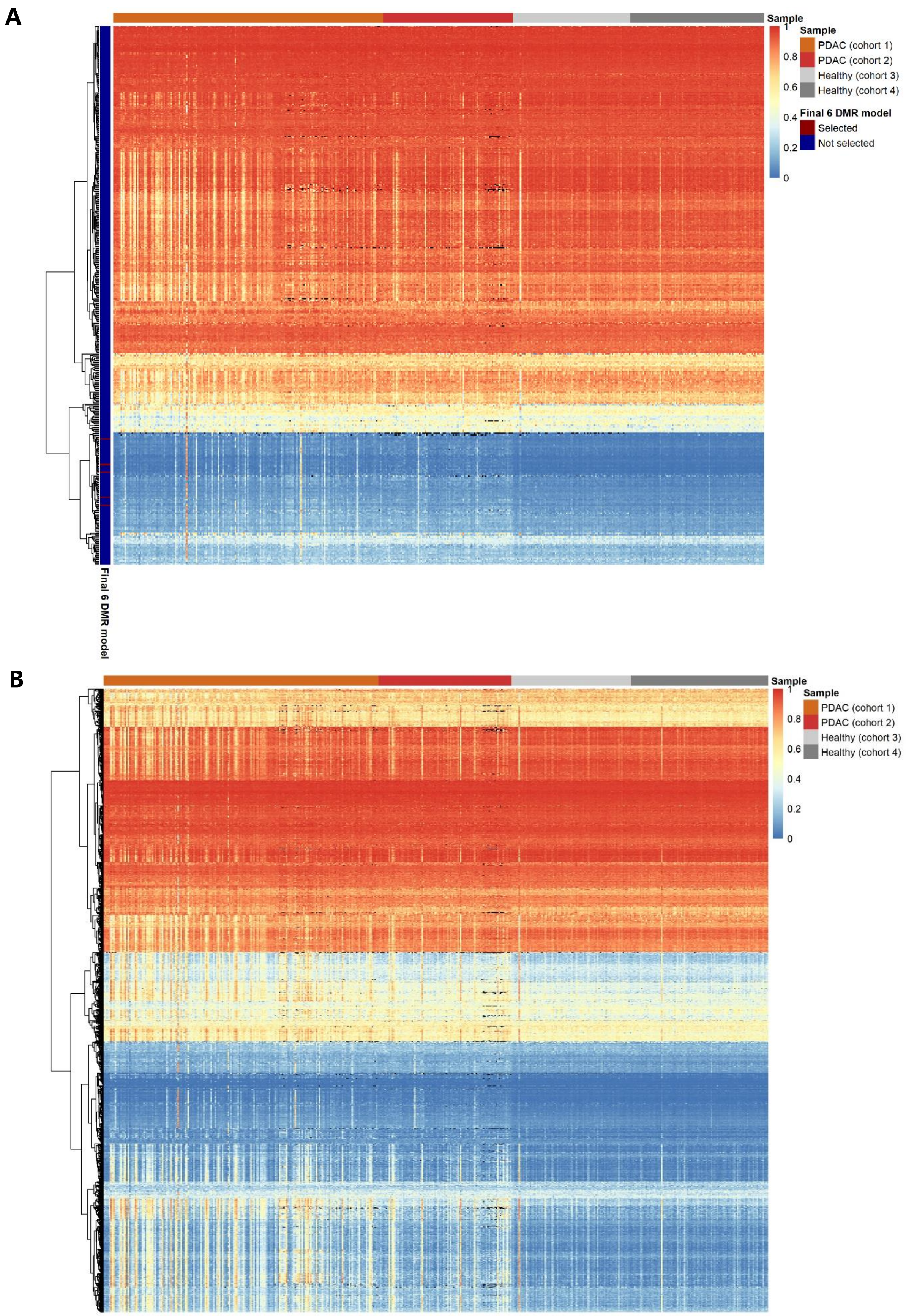

Figure S8. Heatmaps showing methylation levels of 1173 DMRs in plasma cfDNA from PDAC and healthy controls, along with hierarchical clustering of DMRs. Block colors represent the methylation level. (*A*) Hyper-DMRs. (*B*) Hypo-DMRs.

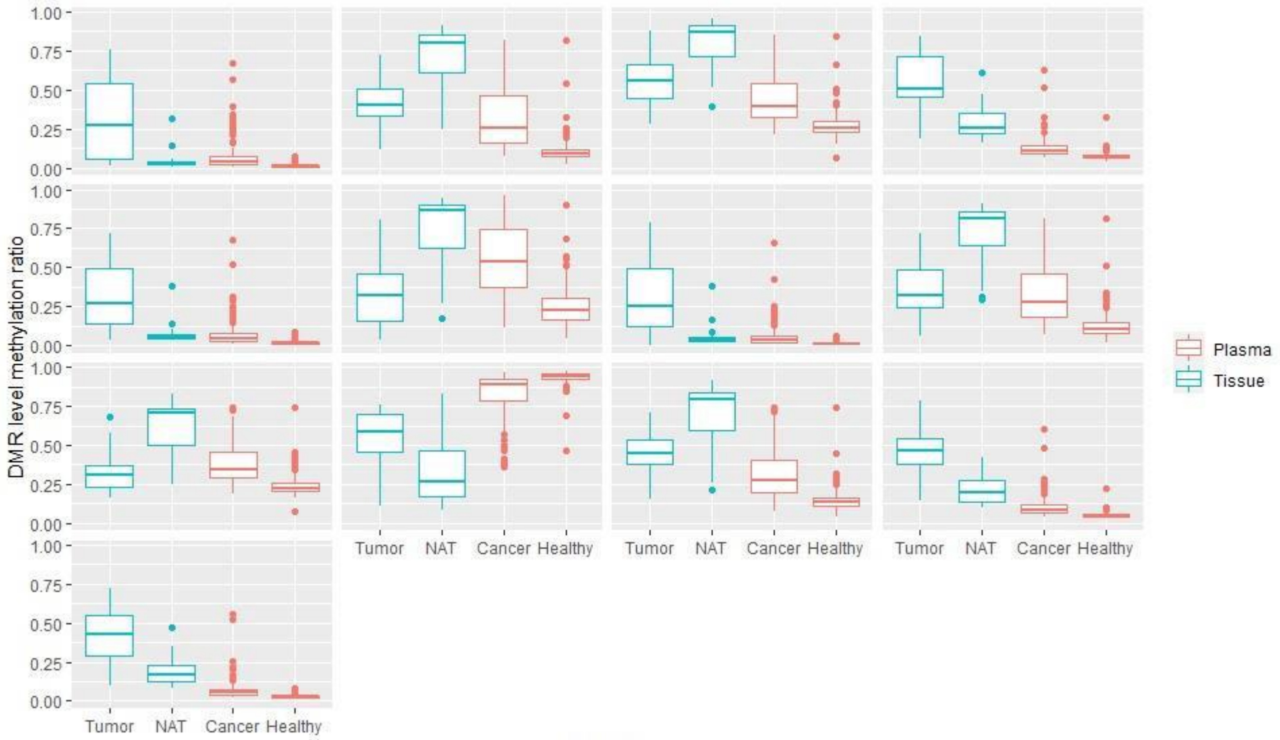

Figure S9. Boxplots showing distributions of cfDNA methylation levels for the 13 DMR markers identified by RFE in the feature selection process.

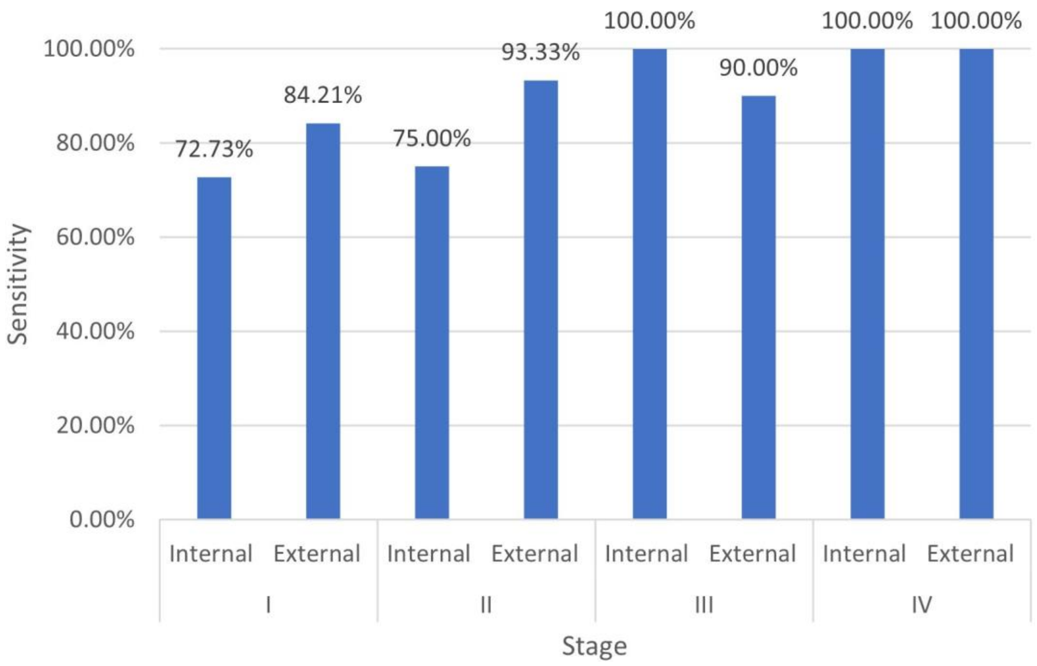

Figure S10. Sensitivity of methylation-based model in internal and external PDAC patients in testing set, respectively.

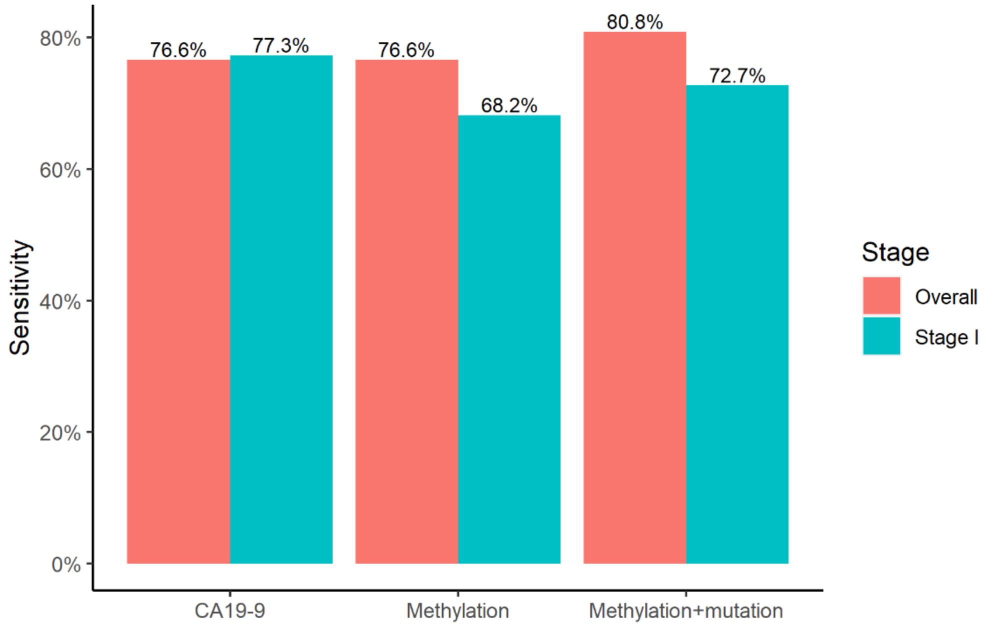

Figure S11. Sensitivity for PDAC patients in testing set by different diagnostic models.
